# Using ‘appreciative inquiry’ in India to improve infection control practices in maternity care: a qualitative study

**DOI:** 10.3402/gha.v8.26693

**Published:** 2015-06-26

**Authors:** Bharati Sharma, K.V. Ramani, Dileep Mavalankar, Lovney Kanguru, Julia Hussein

**Affiliations:** 1 Department of women's and children's health, Karolinska Institute, Stockholm, Sweden; 2 Indian Institute of Management, Ahmedabad, India; 3 Indian Institute of Public Health, Gandhinagar, India; 4 Immpact, University of Aberdeen, Aberdeen, UK

**Keywords:** India, infection control, sepsis, maternal health, maternity services, appreciative inquiry

## Abstract

**Background:**

Infections acquired during childbirth are a common cause of maternal and perinatal mortality and morbidity. Changing provider behaviour and organisational settings within the health system is key to reducing the spread of infection.

**Objective:**

To explore the opinions of health personnel on health system factors related to infection control and their perceptions of change in a sample of hospital maternity units.

**Design:**

An organisational change process called ‘appreciative inquiry’ (AI) was introduced in three maternity units of hospitals in Gujarat, India. AI is a change process that builds on recognition of positive actions, behaviours, and attitudes. In-depth interviews were conducted with health personnel to elicit information on the environment within which they work, including physical and organisational factors, motivation, awareness, practices, perceptions of their role, and other health system factors related to infection control activities. Data were obtained from three hospitals which implemented AI and another three not involved in the intervention.

**Results:**

Challenges which emerged included management processes (e.g. decision-making and problem-solving modalities), human resource shortages, and physical infrastructure (e.g. space, water, and electricity supplies). AI was perceived as having a positive influence on infection control practices. Respondents also said that management processes improved although some hospitals had already undergone an accreditation process which could have influenced the changes described. Participants reported that team relationships had been strengthened due to AI.

**Conclusion:**

Technical knowledge is often emphasised in health care settings and less attention is paid to factors such as team relationships, leadership, and problem solving. AI can contribute to improving infection control by catalysing and creating forums for team building, shared decision making and problem solving in an enabling environment.

Infections due to complications of childbirth are one of the most common causes of maternal and perinatal mortality following childbirth. Sepsis accounts for as much as 15% of the 289,000 maternal lives lost globally every year (1). The prevention of infection is reliant on complex factors (2–4) which include the skills and motivation of health providers, access to drugs and supplies, information feedback, and other building blocks of the health system (3).

Due to the size of its population, India accounts for 19% of the annual maternal deaths worldwide (1). The estimated maternal mortality ratio in India for 2012 was 178 deaths per 100,000 live births (5). Sepsis may be responsible for nearly 20% or around 23,000 annual deaths of all maternal deaths (6). A prospective observational study of 1,016 newborns followed for 28 days, found 48% newborns with serious health problems, 17% had neonatal sepsis with a case fatality rate of 18.5% (7).

Infection control is a vital part of the current national policy for promoting safe births in health facilities and reducing maternal and newborn mortality in India (8). In Gujarat, the government started a hospital accreditation system in 2009 through the National Accreditation Board for Hospitals (NABH) in an effort to improve the quality of care generally, with infection control factors incorporated. The accreditation process involves implementing improvements in management systems including human resource management, supply, finance, information systems, and service quality. In practice, there is still room for improvement in implementing infection control practices in the Gujarat State. The absence of written protocols for infection control and inconsistent adherence to infection control practices, such as reusing gloves, and disinfecting the labour rooms in health facilities offering childbirth services has been documented (9).

The objective of this study was to explore the opinions of health personnel on health system factors related to infection control and their perceptions of change in a sample of hospital maternity units in Gujarat. The ‘change’ introduced was based on findings of a needs assessment in Gujarat which suggested that potential challenges in improving infection control included lack of technical information but were also related to factors such as motivation, problem solving, leadership, and teamwork (9). Appreciative inquiry (AI) is a motivational organisational change process which has been used to overcome implementation barriers, so we postulated that a pathway that used AI could introduce change for improved infection control (Fig. 1). The pathway involves identification of challenges so that processes (e.g. to raise awareness about the problem of infection, take leadership, highlight roles, motivate) could be fostered for action to achieve the goal of improving infection control. Technical inputs would be provided on infection control. We expected that common solutions would emerge, such as change in practices, set up of infection control committees, and standardisation of practices and procedures. Many of these multifaceted aspects of infection control were related to the need to strengthen health system factors or its ‘building blocks’ such as leadership, the delivery of services, equipment, the health workforce, information systems, and resources (3).

**Fig. 1:**
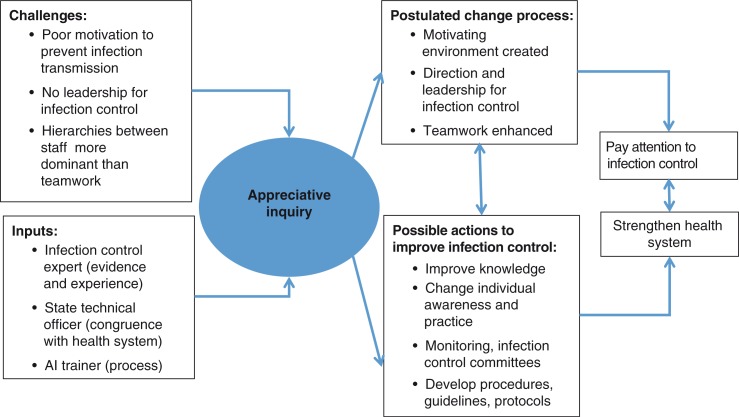
Framework to explore changes in infection control through appreciative inquiry.

In conducting this study, our intention was to better understand how AI could contribute to enhancing infection control within maternity care. AI has been used to enhance management processes since the 1980s (10). Its intention to generate an environment of creativity and learning as part of organisational change (11) is appropriate to complex and systems-related challenges such as infection control. An added advantage of AI is that it introduces change and development by focusing and building on positive aspects, that is, what is done well, so has potential to influence motivational factors. AI has been used in other studies which examine changes to systems of care and behaviour, for example in general practice, nursing, and midwifery in industrialised countries (12–15). There have been reports of its use in service enhancement and planning for maternity care in low and middle income settings (16, 17). We have not found any published work specifically on the use of AI for infection control in low- and middle-income settings.

## Methods

### Setting

The study took place in six secondary and tertiary hospitals in Gujarat. The state has a population of about 60 million with an increasing proportion of women going to hospital for births from 55% to over 70% in recent years (18). The formal health care system comprises government, private for profit, and private non-profit health facilities in four main levels: primary, secondary, tertiary, and, speciality and medical college hospitals at the apex. There are about 500 hospitals in Gujarat where delivery care is generally provided by specialist obstetricians, general medical officers, or nurses. Services in government hospitals are free of charge. In the private non-profit hospitals, services and medicines may be subsidised. Some also have innovative payment schemes to suit the needs of the patient such as paying in instalments.

This qualitative study comprised one component of a wider research project. The second quantitative component monitored post-partum infections in 8,124 women, reported in another paper (19). The selection of six hospitals was based on the number of deliveries required for the quantitative study including sufficient case load (numbers of deliveries and capacity to deal routinely with obstetric complications), mix of government and private non-profit facilities, willingness to participate, and also other practical considerations like feasibility for follow-up.

### Study design

**Fig. 2:**
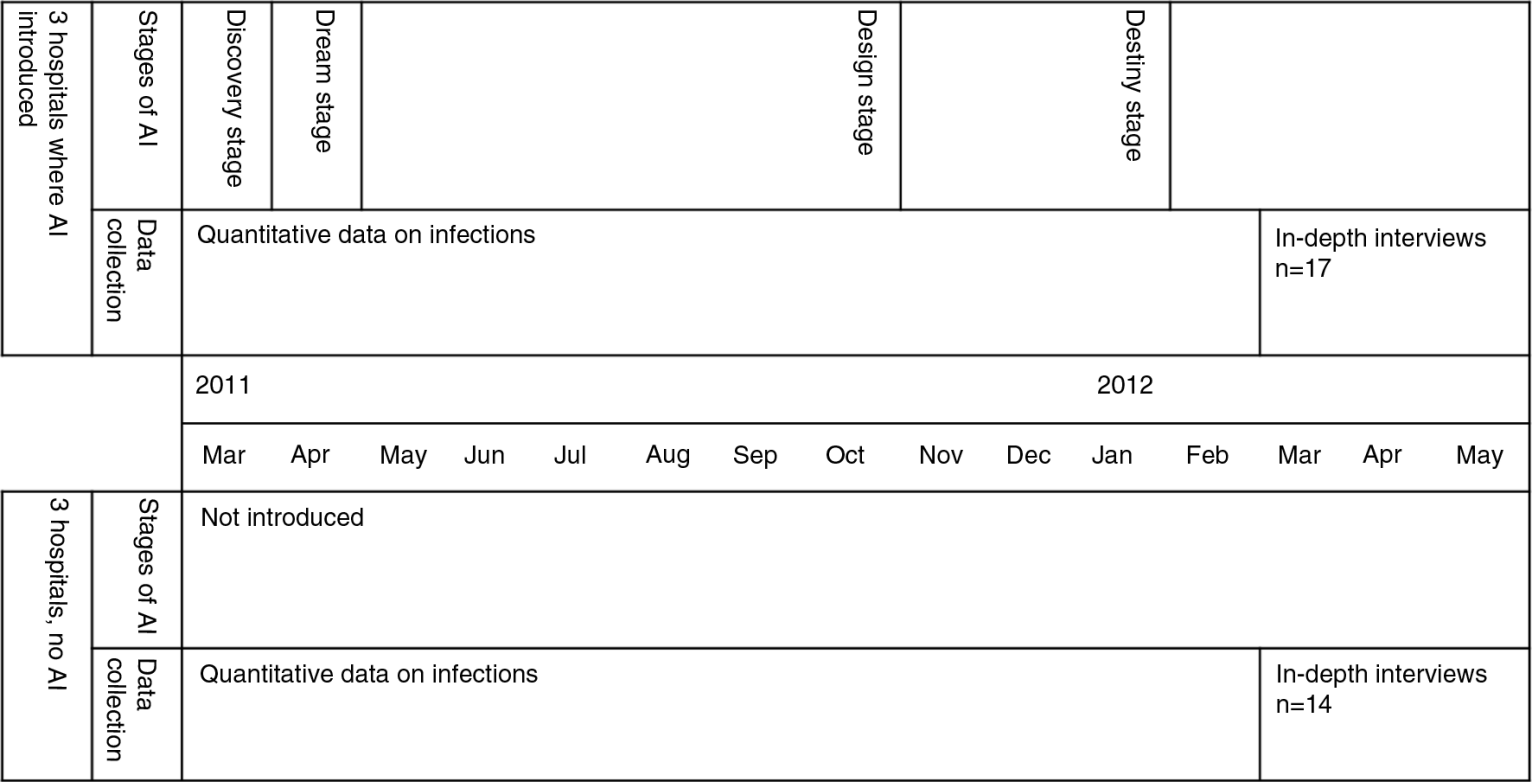
Study Design.

A qualitative approach was used as a means to explore the opinions of health personnel in all six hospitals on their practices, the organisational and physical environment they operated within, and changes they observed. The six hospitals selected were paired according to case load and private or government status and the pair randomly selected to implement AI or not. Three implemented AI (H1–H3) and three did not (H4–H6). In-depth interviews were used and took place between March and May 2012, between 3 and 6 months after the four stages of AI were completed (in the three hospitals). The study design is summarised in Fig. 2.

Of the six hospitals included in the study, there were two government hospitals and one private, non-profit hospital in each group. All the hospitals were rural except for one urban teaching hospital. There were 6–14 nurses and 2–5 doctors employed in the maternity units of the hospitals which conducted between 650 and 4,920 deliveries each year. All hospitals indicated that they had poor patients as their clients.

### Participants

The profile of participants interviewed is summarised in Table 1. Superintendents or heads of hospitals included state-level officers or clinical managers. Practising clinicians of various types, pharmacists, staff nurses, and support staff such as cleaners and messengers were also purposively selected to represent perspectives from a range of health personnel involved in infection control. A total of 31 respondents were interviewed, out of which almost 50% were women, most of them staff nurses and support staff (Table 1).

**Table 1:** Details of key informants

	Hospitals where AI introduced		Hospitals, no AI		
			
Key informants	Male	Female	Male	Female	Total
Superintendent/head	3	0	2	0	5
Medical officers (general physicians)	2	0	1	1	4
Gynaecologists/other specialists	1	0	1	0	2
Staff nurses	0	5	1	4	10
Support staff (general duties and cleaning)	1	2	1	1	5
Pharmacists	0	3	2	0	5
Total	7	10	8	6	31

## Intervention

We used the so-called 4-D model of AI (15) in three hospitals (H1–H3), comprising the following stages: Discovery, Dream, Design, and Destiny, which maps on to our study framework in Fig. 1.

An expert AI trainer, an obstetrician, assisted by the research team, initiated the process, conducting workshops and helping the hospital teams plan actions. All the AI workshops were held to avoid clinical sessions and when hospital personnel were relatively free. Sessions were attended by most of the hospital staff involved in infection control in the hospitals, usually between 6 and 15 individuals.

**Discovery:** This stage involved discovering what had worked well to build upon for improvement and also to identify challenges (in a positive way). In a 1-day session in March 2011, the hospital personnel involved in infection control gathered to share positive experiences of saving women's lives during childbirth. Staff from different hierarchical levels interviewed each other and returned completed interview forms to the trainers.

**Dream:** A stage to develop a vision of where one wants to reach, with postulated changes. In each hospital, a 2-day workshop was held in April 2011. Feedback from the completed interview forms was given, infection control discussed and staff members articulated aspirations and ways to be recognised as ‘The Best Hospital for Infection Control’.

**Design:** A stage to develop a strategy to realise the vision/dreams through actions. Measurable, doable action plans were drawn up. Each hospital formed three or four teams to plan and monitor planned tasks. Regular meetings were held with the research team and state/district health officers for guidance and to solve management challenges. A workshop to review progress, provide technical sessions on infection control (from a specialist) was held.

**Destiny:** This fourth stage was to evaluate achievements and effects of actions on better infection control. Between November 2011 and January 2012, the AI trainer visited each hospital to help to discuss and identify effects and sustainability of the improvements made in each hospital.

The three hospitals where AI was not implemented continued to function as per their usual practice. Researchers visited all six hospitals regularly to collect data on infection (for the quantitative component).

### Data collection

A topic guide was designed to probe domains covering components of the World Health Organization (WHO) health system framework (3). These included issues related to: leadership and governance, which explored hospital-level mechanisms set up by the leaders within each unit, local policies relevant to infection control, autonomy and decision making; availability and quality of services and medical supplies relevant to infection control in maternity wards; health workforce, exploring issues of availability, workload, motivation; and information systems. This conceptual framework (Fig. 1) was used to further probe responses in addition to and within the broader WHO health systems framework.

The interviews were conducted by a male and a female public health professional who were involved in the project since its conception. The male interviewer had past experience in conducting in-depth interviews. Both the interviewers were given a 1-day training by the principle investigator (the last author) regarding ethics involved in interviewing, to familiarise them with the questions, and the important probing questions, as well as the method of asking questions. The interviews were conducted in Gujarati or a mix of Gujarati and English. The interviews took place at the workplace of respondents in a quiet area when they were free from clinical duties. The interviews usually took between 30 min to 1 hour. Interviews were tape recorded and transcribed verbatim by a bilingual researcher (the first author).

During the individual interviews, participants in the six hospitals were first asked to describe the overall situation in relation to infection control in their hospitals, recalling past situations for up to a year. Questions related to health system components with regard to constraints, problems and suggestions for improvement were included. In the second part of the interview, participants were asked to recall any changes they noted recently and within the last 6 months. Respondents were asked to identify differences in practices observed after the study commenced, interpersonal relationships within teams, or their own attitudes and behaviour.

### Data analysis

The analysis was guided by the WHO health systems framework and the theoretical framework (Fig. 1). The interviews were analysed using largely manifest content analysis (visible, obvious components), but also some latent content analysis (interpretation of underlying meanings and relationships) (20) to analyse the interviews. The transcripts were read and re-read to identify ‘meaning units’ (text with similar meaning or describing similar issues across interviews). Thereafter, the meaning units were condensed and grouped into codes, themes, and subthemes. We initially used N Vivo 7 software for identifying meaning units and developing codes. However, N Vivo 7 could not be used for further analysis, as the software did not support the Gujarati language to generate queries. Microsoft word was used to develop themes further and to describe and compare findings. The reported themes were developed and revised through discussion with all the authors and further searching of transcripts done where required.

### Ethical clearance

The Research and Publication Committee of the Indian Institute of Management, Ahmedabad, provided ethical clearance for the study. Permission for the study was granted by the Government of Gujarat.

## Results

Results are from the qualitative study and quantitative findings are reported in another paper (19). Findings are presented, firstly, as opinions and descriptions of the general situation which included the challenges faced, and secondly, as perceptions of changes which might have occurred as a result of AI.

### Descriptions of the general situation and challenges identified

Three themes emerged which provided descriptions of the overall context and challenges faced: management processes, including how hospital accreditation had been carried out, human resource constraints, and physical infrastructure.

#### Management processes

Hospital status in relation to the Gujarat government's and other hospital accreditation schemes were described (Table 2). One hospital (H3) had obtained accreditation from the International Organization for Standardization (ISO), an international network which provides accreditation for quality of services. H5 was in the process of obtaining NABH while H6 had already been given NABH accreditation. Respondents from H3, H5, and H6 described how in aiming for NABH accreditation, all departments had undergone reviews and assessments of strengths and weaknesses, joint planning, goal setting based on the review, allocation of resources, team preparations, periodic capacity building, effective implementation, daily monitoring of services provided, supportive supervision, developing functional information systems, and improving quality of services including patient satisfaction. The staff of H3, H5, and H6 reported improvements in general management systems associated with the accreditation process. For example, hospital H5 had improved waste management, orientation systems for new staff, and refresher training. Specified teams or individuals were assigned to supervise and monitor clinical services and hospital acquired infections and to assure supply of drugs, equipment and other commodities. Although all hospitals kept records and registers of births and related obstetric information, post-partum infections were not routinely monitored in any, except in H5 where infections were monitored as a result of the NABH accreditation process.

**Table 2:** Summary of hospital characteristics

	Hospitals where AI introduced			Hospitals, no AI		
		
Type of hospital and accreditation status	H1 Government	H2 Government	H3 Private, ISO	H4 Government	H5 Government NABH in process	H6 Private NABH
Size and design of physical space						
Adequate	No	Yes	Yes	No	No	Yes
Water supply						
Adequate infrastructure	Yes	Yes	Yes	No	Yes	Yes
Regular	Yes	No	Yes	No	Yes	Yes
Electricity						
Uninterrupted	Yes	No	No	No	Yes	No
Contingency available	Yes	No	Yes	Yes	Yes	Yes

ISO=International Organization for Standardization; NABH=National Accreditation Board for Hospitals.

Hospitals varied in the way decisions could be made. The degree of autonomy which could be exercised at hospital level was limited in some hospitals. Government hospitals generally operated through standard norms with centralised processes for recruitment, transfers of staff and hospital maintenance, which made decision difficult on expansion, reorganisation of infrastructure or hiring staff. For example, the hospitals waited for the state government to deploy staff for vacant positions. In case water or electricity supplies were interrupted or equipment broke down, only pre-specified government agencies could be contacted. Any changes in practice had to be justified. Mismatches between government supplies and facility needs were reported as a result of these norms.

… for example I have been given this monitor and ventilator in the operation theatre by the state government …. We do not have a specialist of medicine (physician) to use this ventilator. Here we get more delivery cases so we should be given more labour tables, more delivery sets …. (Staff nurse H1)

In contrast, the heads of the two private hospitals (H3 and H6) indicated that they take policy and management decisions, which allowed their hospitals to adapt to any immediate needs.

Some hospitals described the use of staff meetings for problem solving and to help in decision making and planning. This process was described in two private hospitals but not in government facilities. Meetings were held for planning, work allocation, monitoring of performance and management control. Respondents described listening to colleagues as team members, identifying challenges and finding solutions collectively.

Q: Do you have staff meetings?

A: We have staff coordination meetings, at the end of each month, a separate meeting for medical stores, and other departments also. We get a chance to put up our problems during these meetings.

Q: Do your problems get resolved?

A: Definitely and many times our suggestions get implemented. If Sir feels what we are saying is logical changes are made … (Pharmacist H6)

A common complaint was that the coordination of the support staff was difficult and not clearly under the supervision of others responsible for the hospital wards:

They come and go as per their wish …. (Staff nurses of H4 and H6)

#### Human resource constraints

Respondents cited shortages of human resource in all cadres across all hospitals. The greatest demand was for doctors or nurses and for support staff such as ward orderlies, ayahs (cleaners) and peons. For example, H1 reported having only two medical officers against the available five posts and three staff nurses against seven posts.

Respondents from the government hospitals said that they coped with staff shortages through ad hoc arrangements approved by the state government such as hiring private services by the day/hour, or multi-tasking by the existing staff. For instance, literate support staff in H1 were deployed to register out patients. In H2, the peon (a local term for support staff who clean and perform errands and other miscellaneous jobs) did general cleaning, dressed wounds, and assisted in surgery as well as deliveries:

… the thing is we have not been assigned specific duties. For example I may be in the dressing section and Sir (doctor) asks me to come to the operation theatre then I have to leave what I am doing and go. In between dressing if there is a delivery case then I have to go to the labour room …. We sweep and mop the floors of the hospital in the morning, after that nothing is fixed …. (Support staff, H2)

During emergencies, doctors and other staff were brought in from other facilities for short periods. The private hospitals also faced shortages of doctors, but could vary their employees according to needs. They trained their own nurses through a short course using their own selection criteria. Multi-tasking was used by the private hospitals too but was limited to doctors and nurses.

Difficulty in finding people to work in cleaning and sanitation was articulated. The complaint was that the job was monopolised by one sector of society and there was protection of colleagues as individuals belonged to a group (a specific community of people defined by caste) which knew each other well:

One of the toughest challenges is sanitation because it is very difficult to get people to work in that department. You may be aware that there is so much monopoly in that business – because it is controlled by only one community. If you hire five people, you cannot fire anyone of them. If you fire one, no one will come from that community. So taking work from them is tricky …. (Superintendent, H6)

Refresher training for specific areas such as infection control was not a regular practice except in H5. Continuing education consisted of orientation to new national or state programmes and occurred mostly in the government hospitals. The support staff were rarely included in any kind of capacity building. In the hospitals which implemented AI, apart from training provided as part of the study, none of the staff had any prior formal training on infection control since their basic entry qualifications. The two private hospitals included some informal capacity building during staff meetings. In the absence of established systems to orient new staff in all hospitals, the new staff learnt tasks by observing other senior staff.

#### Physical infrastructure

The physical infrastructure was a challenge commonly highlighted by respondents. Participant's responses are summarised in Table 2 in three areas especially relevant to infection control: physical space (size of the labour rooms, maternity wards and clinics in relation to the patient load), availability of 24-hour water supply (through taps from mains and boreholes) and electricity supply (including use of generators). Suboptimal situations were described across all hospitals and in all three areas. Staff nurses from H1 mentioned that they had to occasionally put two women on one bed. Although extra beds could be provided, there was no space. This hospital had particular problems as it was originally a primary health centre. The respondents from private hospitals said they had the flexibility and funds to expand and reorganise their space, for example one used a multipurpose hall and several extra beds to prepare an extra ward in emergencies.

All but one hospital had the infrastructure for drawing, storing and supply of borehole water with potential for 24-hour availability on tap. The supply of water was irregular in H2 because of frequent breakdown of the underground bore well and their dependency on other government departments for its repair. H2 faced regular electricity cuts for several hours once a week. They had a generator but only for the blood storage unit. H6 faced regular electricity cuts but installed a generator providing electricity which covered additional areas like residential quarters for staff.

### Perceived changes after introduction of AI

Reported changes and actions taken after introduction of AI are organised in four headings: cleanliness, infection control practices, management processes, teamwork and motivation. Perceived changes are summarised in Table 3.

**Table 3:** Perceptions of respondents on changes related to infection control

	Hospitals where AI introduced			Hospitals, no AI		
		
	H1	H2	H3	H4	H5	H6
**Cleanliness**	Improved	Improved	No change^a^	Improved	Improved	Improved
**Infection control practices**						
Staff compliance in following aseptic practices	Improved	Improved	Improved	Improved	No change	Improved
Regularity in sterilising facilities and equipment	Improved	Improved	No change	Improved	No change	No change
Ensuring patient safety in clinical procedures	Improved	No change	Improved	No change	Improved	Improved
Staff knowledge on infection control	Improved	Improved	Improved	No change	No change	No change
**Management processes**						
Regular meetings	Yes	Yes	Already in place, infection focus introduced	No	No	Already in place, no change
Information system	No change	No change	Improved	No change	No change	Improved
**Teamwork and motivation**	Improved	No change	Improved	No change	No change	No change

a No change indicates that the old system continued or no evidence of change could be elicited from transcripts.

#### Cleanliness

Respondents from five hospitals perceived an improvement in cleanliness in the general hospital premises related to changes in regular dusting of surfaces, more frequent wet mops of floors and more frequent cleaning of toilets. The change was reported not only in hospitals which had undergone AI, as general cleanliness was perceived to have also improved in the other hospitals. A staff nurse from H4 attributed this to the regular visits of the research team for monitoring of infection rates. Respondents in one hospital (H3) said they had not noticed any change in general cleanliness although it should be noted that this was a private, ISO accredited hospital.

#### Infection control practices

Respondents perceived that specific practices had improved after AI. The changes took place differently in the hospitals. In H1, reported improvements in staff compliance included regularity of hand washing, use of gloves, masks and hospital gowns, and changing footwear between operation theatres and labour rooms. H2 began using disposable gloves only once. H3 reported increased use of gloves and changed hand washing practices to include use of a disinfectant. Sterilisation practices also improved. H1 and H2 set schedules or began strictly implementing procedures for fumigation of rooms and for autoclaving equipment. Changes such as using separate baby feeding tubes for each baby and cleaning of baby warmers with antiseptic were reported. Protocols began to be used, for example:

There have been many changes, for example the practice was to note the instructions by the doctor for sick new-borns put in the warmers, on a piece of paper. Now there is a standard sheet for instructions and documentation of care given put up on the warmer itself for everyone to see and follow …. (Staff nurse, H3)

Staff knowledge on infection control was said to have improved in the three hospitals implementing AI.

Challenges remained despite the AI process. Improvements in physical infrastructure were not reported, for example, elbow taps in the labour rooms were not installed despite being identified as a problem. Guidelines and protocols for infection control were not developed widely. The few guidelines for infection control practices such as hand washing technique, and waste disposal systems were not uniformly available. The re-use of gloves did not stop completely because of heavy workloads and short supplies. There were no records for monitoring safe re-use of the gloves.

We need four pairs of gloves per birth (one pair each for the doctor, nurse and 2 servants) and if there are 7–8 births in a day then we need so many gloves … therefore we have to re-use after washing them with antiseptic solution and sterilizing them in autoclave if there is time … (Support staff H2)

Some improvements were seen in the hospitals which did not receive the AI intervention. For example, use of disposable gloves improved in H4 and cleaners were given aprons in H6. One hospital (H4) shifted from boiling to autoclaving labour room equipment. However, fewer changes were noted and respondents said that they perceived no change in staff knowledge on infection control.

#### Management processes

The practice of regular staff meetings was introduced as part of the AI process and was new for the government hospitals. Overall, respondents in all three hospitals which implemented AI said there was benefit from the regular staff meetings. For example, hospital H2 began to discuss each infection case to improve infection control practices. The private hospital (H3), which had a history of regular meetings, also benefitted by focusing discussions more effectively on infection control.

Increased transparency and collective ownership in the organisation, specifically as a consequence of AI, was elicited. In one hospital there was evidence of change to a more approachable leadership:

Today if any staff member notices some objectionable happening which needs to change … they approach me without hesitation. (Head, H3)

This change was reflected in comments from other members of staff not in a leadership role, who indicated that they had begun to discuss mistakes directly with the lead and felt they could do so without fear of reprimand.

Changes were not reported only in the AI sites. A manager of one of the hospitals which did not implement AI described increasing awareness of the need to put into place activities for infection control:

One thing which I learned … and accept is, we should have at least once a year revision or strengthening of these practices through either a workshop or an internal arrangement and we should have still better practices of cleaning and disinfection …. (Head H6)

Health information systems reportedly changed in one hospital implementing AI and one hospital which did not. In H3, post-partum infections were added as an indicator in recording systems. The hospital also introduced post-partum follow-up for 42 days for each mother through telephone (an activity initiated by the research study). In H6, puerperal sepsis was added to the out-patient ‘diagnosis’ list and regular post-partum follow-up and monitoring of infections was commenced, reportedly as a result of increased awareness from participating in the project as a whole.

#### Teamwork and motivation

Staff members described improved team relationships and attitudes after experiencing AI:

because of the [AI] training, we have started to sit and discuss and are able to get things done without hurting anyone … our personality has changed … (Staff nurse H3)

After AI there was motivation for delivering good quality services amongst our staff (Pharmacist, H3)

AI reportedly had a positive influence on the performance of the support staff in particular. Reported reasons included better work allocation and definition of responsibilities, enhanced knowledge about causes of infection and the role of cleanliness, improved self-esteem from the recognition of being part of a team which in turn motivated better performance. The AI sessions and meetings were thought to have improved understanding and appreciation of other's roles:

the staff members understand each other better and have stronger relationships … because of which there is more cooperation and therefore work is accomplished faster. (Pharmacist H3)

It also appeared that improved attitudes in staff had resulted in improvements while interacting with patients. Communication between hospital staff and the patients was also felt to be positively influenced, with development of sensitivity for the disadvantaged backgrounds of the mothers and empathy. One staff member remarked that there was:

… more humaneness in the staff's behaviour towards patients. (Staff nurse H1)

No changes related to teamwork and motivations were reported in hospitals not implementing AI. A perspective from H6 depicted a sense of separation between doctors and other staff (‘them’ referring to nurses and other hospital staff):

When you are a doctor and you are hammered throughout 6 years of education to wash hands, for you it might become your nature, but not for them. (Head H6)

## Discussion

This study provided insights into the key challenges faced in improving infection control in a sample of hospitals in Gujarat and how AI might have contributed to changes for improved infection control. Respondents articulated challenges related to management, including decision making and problem solving modalities, human resource constraints and physical infrastructure (e.g. space, water, and electricity supplies). Our findings suggest that AI may have changed management processes by instigating regular meetings, catalysing discussion and exchange of information so that team relationships were strengthened. There were perceptions that changes affected the actions of health personnel positively and improved infection control practices. This sequence can be related to our postulated pathway of change (Fig. 1) at a rudimentary level. Perceived cleanliness changed in hospitals implementing AI as well as those that did not; in the one hospital where no change was perceived it may be that the accreditation process had already improved standards of cleanliness. Other factors such as regular visits by researchers and collection of data could have confounded the findings; furthermore, the ‘perception’ of cleanliness is likely to be imprecise and subjective.

Controlling infections in health facilities involves following standard practices and technical guidelines which are well established and evidence based (21, 22). Although apparently simple, the technical knowledge is only one part of a complex construct involving multiple domains such as individual attitudes, work values, interpersonal relations, an enabling environment (in the form of supervision, information, supplies), and so on, which has resulted in understanding that multipronged approaches are needed to improve infection control (23–25). We chose AI as our intervention as it has been used as a method of organisational development for complex situations (15, 26). The difference from other conventional methods is its focus on how people think as the basis for what people do (27). We theorised that a change in individual behaviour and attitude towards infection control would also occur in a less overt way.

The changes reported by study participants suggested that AI had potential especially to address organisational or management processes locally, which have been widely identified as a need for infection control in low-resource settings (28, 29). Effects on wider health systems issues such as human resources and physical infrastructure seemed to be less amenable to change. Contextual factors were important, for example the restricted autonomy for decision making in government hospitals could weaken potential for change. In any case, system wide issues, common to many low-resource settings (30) are not always solvable at local hospital level, nor are they necessarily possible to change in the relatively short duration of this project. Obtaining accreditation appeared to consist of carrying out management processes which would have been implemented through AI as well, albeit without consideration of building on positive aspects. It was the observation of the researchers that uptake and willingness to take on AI was higher in H3 (an accredited hospital) where the importance of infection control within the wider quality improvement effort was brought out. It is possible that accreditation can put hospitals in a receptive, active mode for change. No negative aspects of AI were reported.

Some findings suggested that covert factors were in play. Within the human resource theme, wider societal factors emerged. India is a setting where cleaning staff are lowest in the caste hierarchy in the Hindu society. Yet they are not entirely divested of power. As illustrated in one of the quotes provided, they have an informal yet strong association supporting each other's interests, which need to be considered in interventions to support and focus on cleaning staff. The staff appeared to have felt the benefits of discussions introduced by AI which allowed them to see their organisation and colleagues with new insights. For the two government hospitals it was a rare occasion when all the hospital staff of different levels came together for discussion. Team relationships, motivation, a sense of belonging and being listened to developed. Improved approachability of the leadership across hierarchal levels was evident. Having committees with representation of all levels of staff from the doctors to cleaners, helped in appreciating the interdependence and inter-connectedness of each member's role. Most work in human resource management for health has concentrated on health professionals and paramedical staff (31–33) and our study has highlighted the special importance of exploring the role of support and cleaning staff in particular for infection control initiatives.

This study was subject to a number of limitations which constrain the interpretation of our findings. We have discussed how accreditation, visits of researchers and the differences between private and government hospitals may have affected the findings. Although we attempted to segregate our findings by individual hospitals (Table 3), it is not possible to disentangle the effects of these and other factors. Our use of the six health system building blocks (3), used as a basis for the topic guide, was driven by the multifaceted aspects of infection control and probably influenced the emergent themes, limiting an inductive and in-depth exploration of some factors such as motivation and dominant hierarchies. These underlying factors complicated our ability to interpret the influence of AI, although we believe there is at least perceived evidence of the direct effects of AI on motivation, attitude and practice. We tried to limit intrusive activities but observer bias was inevitable as study aims were explained and hospital staff knew that data was being collected. This might have led to a prioritisation of infection control. Recall bias was a possibility as we asked the key informants to describe situations as far back as 1 year but this was unavoidable as we did not want the interviews to affect the planned intervention. The number of hospitals and informants participating is small so the findings may not be representative and should be interpreted with caution.

## Conclusion

This study used AI methodology to elicit stakeholder input in a change process focusing on infection control in hospitals, which had a positive influence on infection control practices; Forums were established for team building and enabling shared decision making, problem solving and capacity building. There were several other factors in play which were difficult to disentangle from the effects of AI. Unexpectedly, social factors such as the caste system were re-enacted in teams at the workplace, unearthing further complexities which need to be considered in effecting infection control. Technical knowledge and training are usually at the forefront of infection control initiatives but we demonstrate that attention should be paid to equally important factors of good management practices, team bonding, egalitarian leadership, communications and interpersonal relations. AI is a means for developing a shared ideology and values for improved service delivery, thereby improving organisational ‘work culture’. Some of the findings are relevant to the local context, however the organisational challenges we came across are common to many low-resource settings and AI may be a valuable process to study and implement in other low and middle income countries.
